# The pharmacokinetics and pharmacodynamics of esomeprazole in sheep after intravenous dosing

**DOI:** 10.3389/fvets.2023.1172023

**Published:** 2023-05-05

**Authors:** Joe S. Smith, Jessica Gebert, Kailee Bennett, Lisa Sams Ebner, Ryan Flynn, Pierre-Yves Mulon, Lainey Harvill, Olivia Grace Escher, Amanda Jo Kreuder, Joan Bergman, Sherry Cox

**Affiliations:** ^1^Large Animal Clinical Sciences, College of Veterinary Medicine, University of Tennessee, Knoxville, TN, United States; ^2^Biomedical Sciences, College of Veterinary Medicine, Iowa State University, Ames, IA, United States; ^3^Lincoln Memorial University, College of Veterinary Medicine, Harrogate, TN, United States; ^4^Biomedical and Diagnostic Sciences, College of Veterinary Medicine, University of Tennessee, Knoxville, TN, United States; ^5^Veterinary Microbiology and Preventative Medicine, College of Veterinary Medicine, Iowa State University, Ames, IA, United States

**Keywords:** abomasal ulcer, abomasal ulceration, ewe, gastric, gastroprotectant, proton pump inhibitor (PPI)

## Abstract

Abomasal (gastric) ulceration is a morbidity in sheep, and currently, there is a paucity of pharmacokinetic and pharmacodynamic data for gastroprotectant drugs reported for this species. The proton pump inhibitor esomeprazole has been used in small animal and human patients for gastroprotection via increasing the gastric pH. The objective of this study was to report the pharmacokinetic parameters and pharmacodynamic effect of esomeprazole in sheep after single intravenous dosing. Four healthy adult Southdown cross ewes had blood collected over a 24  h time period after single intravenous dosing of esomeprazole at 1.0  mg/kg. Abomasal fluid was sampled over 24  h before and after esomeprazole administration. Plasma samples were analyzed for concentrations of esomeprazole and the esomeprazole metabolite, esomeprazole sulfone by high performance liquid chromatography. Pharmacokinetic and pharmacodynamic data were evaluated with specialized software. Esomeprazole was rapidly eliminated after IV administration. Elimination half-life, area under the curve, initial concentration (C0), and clearance were 0.2  h, 1,197  h*ng/mL, 4,321  ng/mL, and 0.83  mL/h/kg, respectively. For the sulfone metabolite elimination half-life, area under the curve and maximum concentration were 0.16  h, 22.5  h*ng/mL, and 65.0  ng/mL, respectively. Abomasal pH was significantly elevated from 1 to 6  h after administration and remained above 4.0 for at least 8 h after administration. No adverse effects were noted in these sheep. Esomeprazole was rapidly eliminated in sheep, similar to goats. Abomasal pH was increased, but future studies will be necessary to develop a clinical management approach to the use of esomeprazole in sheep.

## 1. Introduction

Abomasal ulceration is a common cause of morbidity and mortality in sheep of all ages (1–3). While the causes and etiology of abomasal ulcers in sheep are not fully elucidated, risk factors include stressful situations, nonsteroidal anti-inflammatory drug administration, diets high in rapidly fermentable carbohydrates, and other diseases. These factors contribute to a decreased abomasal pH, as well as protection of gastric mucosa. Across ruminant species, treatment of abomasal ulcers include neutralization of acid, prevention of secretion of acid, and protection of damaged gastric mucosa. Comparatively across species, it is thought that achieving a gastric pH above 4.0 can be beneficial for the management of gastric ulceration (4).

Proton pump inhibitors (PPIs) are a main class of anti-ulcer drugs that function via inhibition of the hydrogen/potassium adenosine triphosphate (ATP) pump or “gastric proton pump.” This action of this pump is the last step before secretion of the hydrogen ion into the gastric lumen, and as such PPIs are typically more effective than other gastroprotectant drugs, such as the histamine H2-receptor antagonists, which work further upstream in the process. The PPIs also irreversibly bind to the proton pump, so the action of the pump is terminated until the cells lining the gastric lumen turn over. While monogastric species can be administered PPIs orally, the rumen serves as a barrier by metabolizing and diluting oral medications before they can be absorbed in ruminant species such as sheep, making injectable formulations ideal for clinical use. While there are clinical studies describing the use of PPIs in multiple ruminating species, including sheep, goats, cattle, yaks, and alpacas (5–10), there is limited prospective literature demonstrating the pharmacokinetics and pharmacodynamics of PPIs, such as esomeprazole, in sheep.

Esomeprazole is a substituted benzimidazole (C_17_H_19_N_3_O_3_S) that is the s-enantiomer of omeprazole. It is commercially available in an injectable formulation, which would avoid issues associated with oral absorption. The goal of this study is to describe the pharmacokinetics of esomeprazole and its sulfone metabolite in sheep after intravenous injection. An additional goal of the study was to describe the pharmacodynamic effect of esomeprazole after intravenous injection on the abomasal (gastric) pH in sheep.

## 2. Materials and methods

### 2.1. Animals

This study utilized four adult Southdown cross ewes fitted 6 weeks prior with surgically implanted abomasal cannula as described for calves by Olivarez et al. (11) The ewes were approximately 2.75 ± 0.96 years of age and weighed 67.9 ± 7.6 kg. They were determined to be clinically healthy based on physical examination prior to the study and had no history of ulceration or clinical signs of ulceration. The ewes were housed in individual pens for the entirety of the study period and had been housed in these pens for 8 weeks prior to study implementation. During this time, the ewes were not group housed but were housed in immediately adjacent pens of chain link fence material to allow for social interaction. They had *ad libitum* access to grass hay and water starting 7 days prior to the study. All animal procedures in this study were approved by the Institutional Animal Care and Use Committee (IACUC #2835-0521) of the University of Tennessee.

An intravenous jugular catheter (MILACATH®-EXTENDED USE, 16 Ga × 7.5 cm, MILA International Inc.) was placed aseptically (one in each vein) 2 h prior to initiation of the 24-h study. One catheter was reserved for drug administration, and the second catheter was designated for sample collection. No drugs had been administered to the sheep for 18 days prior to the study. Esomeprazole (Esomeprazole Sodium for injection, Mylan International, Rockford Il) was administered at a 1.0 mg/kg dosage as a single dose (based on a described intravenous dose for goats) (12), with the dosing catheter flushed with 10 mL of 0.9% saline afterwards to ensure all drug was administered.

Blood samples were obtained from the designated sampling jugular catheter at 0, 5, 10, 20, 30, and 45 min after administration. Blood samples were also collected 1, 1.5, 2, 3, 4, 8, 12, 18, and 24 h after administration. Whole blood samples were collected via the push-pull technique from the designated catheter, with 4 mL blood placed into a lithium heparin tube (BD Vacutainer, BD) and immediately put on ice prior to centrifugation. Samples were spun down at 3,000 × *g* for 10 min and then plasma placed in cryovials (Cryogenic Vials, Biologix) and frozen at −80°C.

### 2.2. Abomasal pH measurement

Abomasal fluid was collected via the abomasal cannula as described for calves (11). Fluid was collected each day of the study (day 0: pre-esomeprazole administration “control” and day 1 post-esomeprazole administration) using the same schedule: 0, 1, 2, 3, 4, 6, 8, 12, 18, and 24 h for both control samples as well as after esomeprazole administration. A 3 mm × 70 mm stainless steel two-eyed teat cannula attached to a 12 mL syringe was introduced into the cannula past the one-way valve in the cannula. Fluid was aspirated via negative pressure using the 12 mL syringe until 4–5 mL of fluid was collected. The pH of each sample was then recorded within 15 min of collection. The samples of abomasal fluid were placed into a 30 mL conical vial. A benchtop pH analyzer (UB-10 pH/mV meter, Denver Instruments, United States) was then used to measure pH. The analyzer was calibrated prior to each sample set according to the manufacturer’s procedure. Once calibrated, the probe was introduced into the abomasal content sample and equilibrated for 30 s, which is when the pH level was recorded.

### 2.3. High performance liquid chromatography analysis

Esomeprazole and its metabolite concentrations were detected in sheep samples using a validated method for proton pump inhibitors in goats (12, 13). The system consisted of a 2695 separations module and a 2487 UV absorbance detector (Waters, Milford, MA, United States). Separation occurred on a Symmetry C_18_ (4.6 mm × 150 mm, 5 μm) column with a 5 μm Symmetry C_18_ guard column (Waters, Milford, MA, United States). The mobile phase was a mixture of 20 mM ammonium acetate and acetonitrile (75:25). The absorbance was measured at 290 nm, and the flow rate was 1 mL/min.

Esomeprazole and its metabolite were extracted from plasma samples using a liquid–liquid extraction method. Samples that were previously frozen were thawed at room temperature and vortex-mixed. 100 μl of plasma was transferred to a 13 mm × 100 mm screw top tube, followed by 10 μL of tinidazole (internal standard, 10 μg/mL), and 1 mL chloroform. The mixture was rocked for 10 min and underwent centrifugation for 10 min at 1,000 × *g*. The organic layer was transferred to a glass tube and evaporated to dryness. Samples were reconstituted in 225 μL of mobile phase to insure an adequate volume for injection into the HPLC system, and 100 μL was the volume analyzed.

Method validation was performed based on the Food and Drug Administration (FDA) Bioanalytical Guidelines (14). Standard curves for the plasma analysis were prepared by fortifying untreated, pooled plasma with esomeprazole and its metabolite (analytical standards obtained from Cayman Chemical, MI, United States), which produced a linear concentration range of 5–5,000 ng/mL. Recovery, accuracy, and precision were assessed by analyzing five replicates at low, medium, and high concentrations within the concentration range of the curve. The quality control (QC) concentrations used were 15, 75, 300, 1,300, and 4,000 ng/mL. The accuracy of the assay was within 100, 99, 102, 102, and 101% for esomeprazole and 106, 105, 105, 103, and 103% for the metabolite for the QC concentrations used. The precision of the assay using the QC concentrations was (expressed as CV%) 0.77, 4.09, 2.62, 7.32, and 7.93% for esomeprazole and 5.99, 8.55, 2.47, 3.61, and 7.58% for the metabolite. The recovery for esomeprazole ranged from 99% ± 2 to 101% ± 5, while the range for the metabolite was 99% ± 2 to 103% ± 5 for 15, 75, 300, 1,300, and 4,000 ng/mL, respectively. The average recovery for the internal standard was 99% ± 2. The lower limit of quantification for both was 5 ng/mL. The limit of detection was 2.5 ng/mL.

### 2.4. Pharmacokinetic analysis

Noncompartmental pharmacokinetic modeling was performed from time-plasma concentration data via standard modeling software (PKanalix, Monolix Suite 2021R2, Lixoft, France) as described for proton pump inhibitors in goats and calves (11, 12, 15, 16). Standard PK parameters were generated for individual ewes, as follows: Maximum concentration of esomeprazole extrapolated to time zero, C0; Time of maximum esomeprazole concentration, Tmax; Area under esomeprazole concentration–time curve, AUClast (AUC_0-24h_) and AUCinf; Area under the moment curve, AUMCinf; esomeprazole mean residence time, MRT = AUMCinf/AUCinf; esomeprazole terminal half-life, T_1/2_ (λz) = ln (2)/λz; esomeprazole systemic clearance, CL = Dose/AUCinf; Volume of distribution of esomeprazole (area), Vz. A linear/log trapezoidal rule was used for data analysis to estimate the area under the esomeprazole time-curve. Summary statistics on the individual PK parameters were performed thereafter to derive the geometric mean [harmonic mean for elimination half-life (17, 18)], minimum, and maximum.

### 2.5. Statistical evaluation

Abomasal pH values were screened for normality then evaluated with the appropriate paired T-test by a commercial statistical software program (GraphPad Prism version 8.0.0 for Windows, GraphPad Software, San Diego, California, United States). Each time point was compared to the corresponding time point of the control sampling period. A *p* value of less than 0.05 was considered statistically significant.

## 3. Results

### 3.1. Animals

Abomasal cannulas were well-tolerated by all ewes, with maintenance of appetite and body weight throughout the entire study. Cannulas were removed 3 days after collection of the last samples. Follow up 4 months after the conclusion of the study demonstrated all ewes were in good body condition and were reported as doing well. Esomeprazole administration appeared to be well tolerated with no evidence of adverse reaction after administration.

### 3.2. Abomasal pH measurement

**Figure 1:**
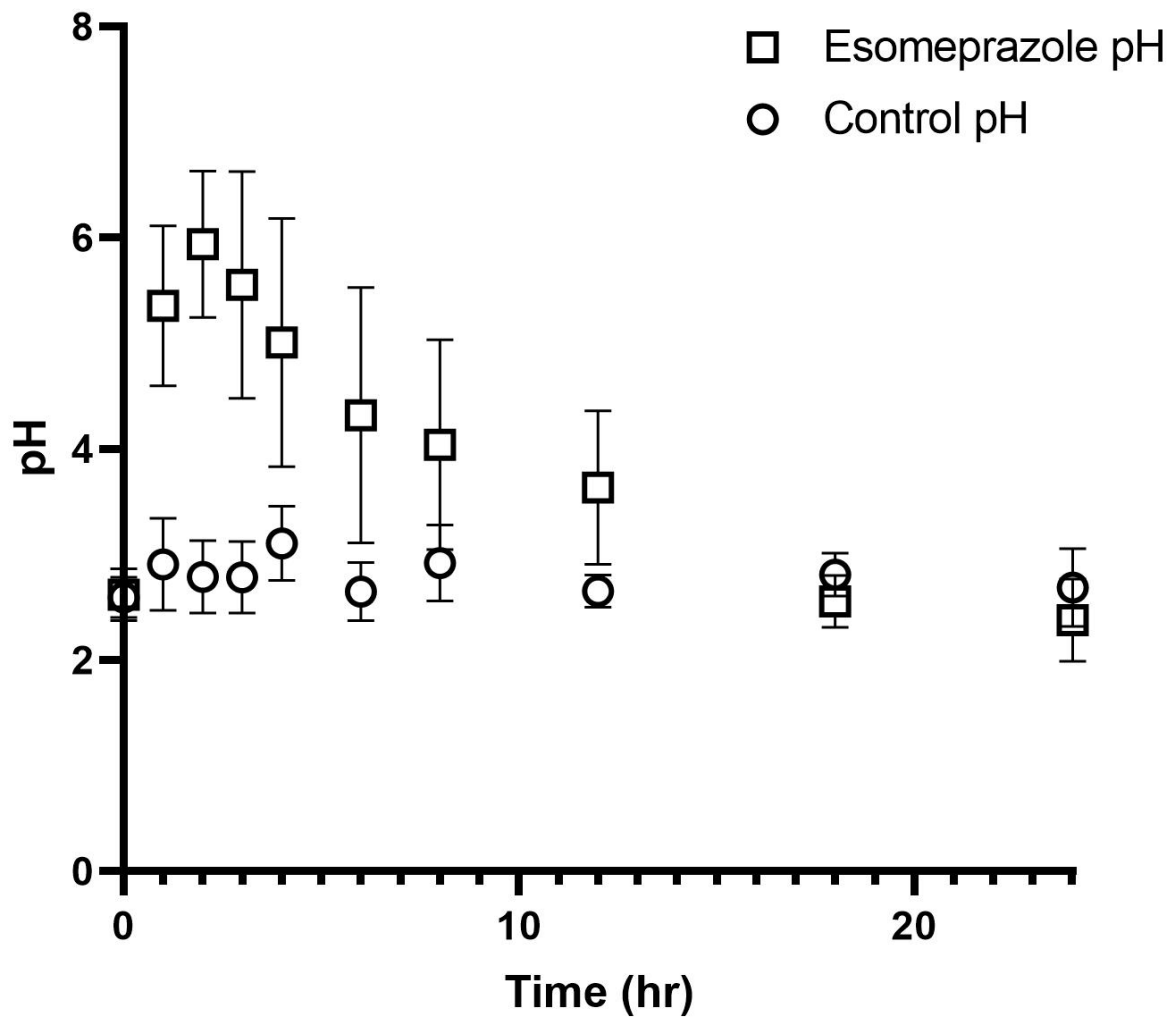
Values for pH before and after intravenous administration of esomeprazole (1  mg/kg) in four healthy adult ewes. Circles represent pH values prior to esomeprazole administration and squares represent pH values after esomeprazole administration.

Abomasal pH on day 0 (control) ranged from 2.59 ± 0.19 to 3.11 ± 0.35. After IV administration of esomeprazole, abomasal pH peaked at a high of 5.94 ± 0.70 at 2 h post-administration before slowly decreasing to a low of 2.38 ± 0.39 at 24 h post-administration. After esomeprazole treatment, statistically significant increases in pH over baseline were observed from 1 to 6 h after administration (Table 1) and remained increased over 4.0 until at least the 8-h time point (Figure 1), as well as over 3.6 until at least the 12 h timepoint.

**Table 1:** Results of abomasal fluid pH from four adult ewes collected for 24  h before esomeprazole administration (Control) and for 24  h after administration of 1  mg/kg of esomeprazole intravenously.

Time (Hr)	Control (pH Mean ± SD)	Esomeprazole (pH Mean ± SD)	*p* value
0	2.59 ± 0.19	2.62 ± 0.25	0.875
1	2.91 ± 0.44	5.36 ± 0.76	**0.0101**
2	2.79 ± 0.35	5.94 ± 0.70	**0.0033**
3	2.79 ± 0.34	5.56 ± 1.08	**0.0122**
4	3.11 ± 0.35	5.01 ± 1.18	**0.0301**
6	2.65 ± 0.28	4.32 ± 1.21	**0.0473**
8	2.92 ± 0.36	4.04 ± 1.00	0.0714
12	2.65 ± 0.15	3.63 ± 0.73	0.0534
18	2.81 ± 0.20	2.56 ± 0.25	**0.0477**
24	2.69 ± 0.37	2.38 ± 0.04	0.2736

A *p* < 0.05 was considered statistically significant (bolded).

### 3.3. Pharmacokinetics

**Figure 2:**
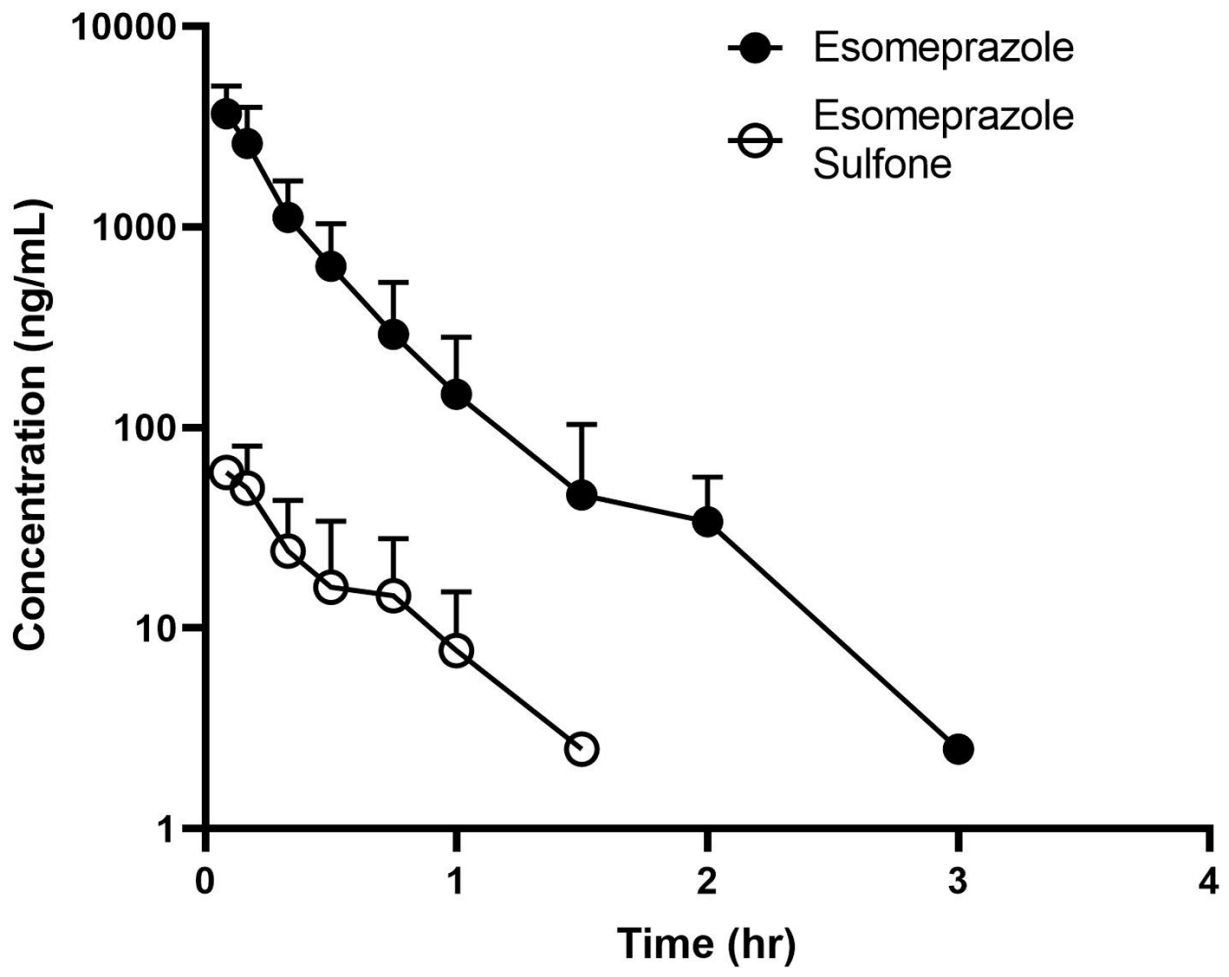
Time vs. concentration data for esomeprazole (solid circle) and the metabolite esomeprazole sulfone (open circle) after intravenous administration (1.0  mg/kg) in four adult ewes.

Time vs. plasma concentration information is displayed in Figure 2. After administration, the parent drug was detectable for up to 3 h, and the metabolite was detectable for up to 1.5 h. Maximum (C0) concentrations were 4,323 ± 777 for the esomeprazole parent drug and (Cmax) 65.0 ± 21.8 for the sulfone metabolite. Pertinent pharmacokinetic parameters are listed in Table 2. Extrapolation % of the AUC was 4.78 ± 1.79%.

**Table 2:** Pharmacokinetic parameters of esomeprazole and the metabolite esomeprazole sulfone after single dose intravenous (IV; 1  mg/kg) administration in adult sheep (*n* = 4).

Compound	Parameter	Unit	Mean* ± SD	Min	Max
Esomeprazole	C0	ng/mL	4,323 ± 777	3,523	5,247	AUC_last_	h*ng/mL	1,197 ± 577	738	1938	AUC_inf_	h*ng/mL	1,198 ± 578	738	1939	AUMC_inf_	h*ng/mL	296 ± 284	115	723	MRT_inf_	h	0.25 ± 0.099	0.16	0.36	Cl	mL/h/kg	0.83 ± 0.32	0.56	1.29	T_1/2_ (λz)	hr	0.20 ± 0.81	0.15	0.29	λz	1/h	3.36 ± 1.32	2.36	4.75	V_z_	L/kg	0.25 ± 0.035	0.21	0.28	Vss	L/kg	0.21 ± 0.015	0.19	0.23
Esomeprazole sulfone	C_max_	ng/mL	65.0 ± 21.8	49	96	T_max_	h	0.099 ± 0.0042	0.083	0.17	AUC_last_	h*ng/mL	22.5 ± 16.0	13.6	49.1	AUC_inf_	h*ng/mL	23.6 ± 16.2	14.5	50.3	MRT_inf_	h	0.23 ± 0.14	0.14	0.45	T_1/2_ (λz)	h	0.16 ± 0.075	0.12	0.28	λz	1/h	4.0 ± 1.59	2.5	5.62

C0, calculated concentration at time zero of IV administration; AUC_last_, area under the curve calculated at the last time point; AUC_inf_, area under the curve extrapolated to infinity; AUMC_inf_, area under the moments curve extrapolated to infinity; MRT_inf_, mean residence time extrapolated to infinity; CL, plasma clearance; T_1/2_ (λz), elimination half-life (harmonic mean); λz, elimination rate; Vz, volume of distribution; Vss, volume of distribution at steady state; Cmax, maximum plasma concentration (sulfone metabolite); and Tmax, time to reach maximum plasma concentration (sulfone metabolite).

## 4. Discussion

In this study, the PK of esomeprazole and its sulfone metabolite are described in adult sheep after a single intravenous administration of a 1.0 mg/kg dose of esomeprazole. Abomasal pH values remained above 4.0 for at least 8 h after administration and were significantly elevated from 1 to 6 h after administration. No apparent adverse effects were noted in any of the ewes after intravenous esomeprazole administration.

Similar to goats, esomeprazole was rapidly eliminated in the sheep. The sheep elimination half-life of 0.2 h is slightly longer, but still very rapid compared to 0.1 h for goats (12). A longer elimination half-life was noted after IV administration in dogs, with a reported value of 0.73 h (19). Initial concentrations in the sheep study were 4.32 μg/mL (4,320 ng/mL) compared to 2.32 μg/mL in goats (12). Area under the curve was higher in dogs (3.82 h*ng/mL) compared to sheep (1.20 h*ng/mL) and goats (0.44 h*ng/mL), indicating higher exposure in canine species than ovine or caprine. Reported veterinary pharmacokinetic parameters for esomeprazole are comparatively presented in Table 3. Other than species-specific differences in drug metabolism, one of the explanations for the difference in pharmacokinetics between dogs and the sheep in our study may be analytical sensitivity. The assay in the canine study had a lower limit of quantification of 0.5 ng/mL, and the sheep study had a lower limit of quantification of 5.0 ng/mL. Different limits of quantifications in assays can alter parameters, such as area under the curve and elimination half-life (20). The sulfone metabolite in our sheep had a higher maximum concentration (65 ng/mL) than reported in goats (32), and the elimination half-life of the metabolite in sheep was faster (0.16 h) than reported in goats (0.63 h), but both species rapidly eliminated the sulfone metabolite (12). In calves administered the similar PPI pantoprazole, the parent drug was not detectable in tissues; however, the sulfone metabolite was detectable in tissues (16). It is currently unknown if this is also true for sheep, but it would be useful information to have for withdrawal recommendations.

**Table 3:** Comparison of the PK parameters reported from the veterinary literature and the ovine subjects of this study.

Species	Dose (mg/kg); Route	C0/C_max_ (μg/mL)	AUC_last_ (h*μg/mL)	AUC_inf_ (h*μg/mL)	Cl (L/h/kg)	T_1/2_ (h)	Vss (L/kg)	Reference
Canine	1.0; IV	-	3.82	3.82	0.3	0.73	0.27	Hwang (19)
Canine	1.0; SC	2.62	4.07	4.07	NA	0.90	NA	Hwang (19)
Caprine	1.0; IV	2.32	0.44	0.44	1.50	0.1	0.23	Fladung (12)
Caprine	1.0; SC	1.04	1.02	1.03	NA	0.49	NA	Fladung (12)
Ovine	1.0; IV	4.32	1.20	1.20	0.83	0.2	0.21	Present Study

C0/Cmax, peak plasma concentration; AUC, area under the plasma concentration-time curve, extrapolated to last time point (AUC_last_) and to infinity (AUC_inf_); Cl, systemic plasma clearance; T_1/2_(λz), terminal (elimination) half-life; Vss, steady state volume of distribution; NA, not applicable. Units converted for uniform presentation.

The effect of esomeprazole increasing abomasal (gastric) pH was appreciated in this study with values being above baseline for a minimum of 12 h after administration. Similar elevations have been noted with other PPIs, notably pantoprazole in alpacas and calves (10, 11). Esomeprazole administration has also been shown to significantly increase the gastric juice pH in horses after administration (21). In equine and human medicine, achieving a gastric pH of >4.0 for greater than 66% of a 24-h period is ideal for the healing of gastric ulceration (4). Our study’s data suggest that IV administration of esomeprazole can achieve this comparatively therapeutic level for at least 8 h, although currently the time needed to be above a certain pH for the healing of gastric ulcers in sheep is not definitively known.

Limitations of this study include the small number of ewes studied. While pharmacokinetic studies can be appropriately powered with 4–6 animals (22), and studies of four animals have been utilized in previous investigations of ruminant gastroprotectants (23), this may not be a large enough study population to capture population variability. An additional limitation is the sampling schedule, which was based on a sampling schedule for milk-fed calves, where there are differences in feeding compared to these sheep on free choice hay. In hindsight, more intense sampling would be useful for complete determination of abomasal pH changes, so that a complete pH timeline could be elucidated as was done previously with pantoprazole in alpacas (10).

Future studies should consider more animals, more intense sampling strategies to better evaluate pharmacodynamic outcomes, and other routes of administration. While other proton pump inhibitors in ruminants have not been demonstrated to have adverse effects associated with their usage (5), adverse effects have been reported with the human use of this drug class, therefore, and further studies should also evaluate the clinical safety of esomeprazole in sheep. Clinical questions to be answered with more investigation would be the efficacy and pharmacokinetics of subcutaneously administered esomeprazole, as in goats subcutaneous administration had a bioavailability of 116% (12), and this route of administration could be beneficial for on-farm use, as well as duration of therapy needed for clinical effect and calculation of appropriate withdrawal times. Non-linear mixed effects (NLME) modeling could also be employed to better understand the relationship between parent compound, metabolite and pharmacodynamics (24).

In conclusion, esomeprazole administered intravenously at a dose of 1.0 mg/kg in adult ewes results in abomasal pH levels above 4.0 for a minimum of 8 h after administration. Pharmacokinetic parameters of esomeprazole in sheep are characterized by rapid elimination, although in these sheep the elimination was longer than reported for goats with similar dosing. While esomeprazole increases abomasal pH for several hours after administration, future studies are needed to determine an appropriate dosing regimen to maintain therapeutic pH values for the healing of abomasal ulceration in sheep.

## Data availability statement

The original contributions presented in the study are included in the article/Supplementary material, further inquiries can be directed to the corresponding author.

## Ethics statement

The animal study was reviewed and approved by Institutional Animal Care and Use Committee, University of Tennessee.

## Author contributions

JS, P-YM, LE, and AK established the study design. JS, P-YM, and KB implemented the cannulation method. JS, KB, JG, LE, RF, OE, and LH contributed to sample collection and study implementation. JB and SC developed the analytical method and performed sample concentration determination. JS and KB contributed to data analysis. All authors contributed to the article and approved the submitted version.

## Conflict of interest

The authors declare that the research was conducted in the absence of any commercial or financial relationships that could be construed as a potential conflict of interest.

## Publisher’s note

All claims expressed in this article are solely those of the authors and do not necessarily represent those of their affiliated organizations, or those of the publisher, the editors and the reviewers. Any product that may be evaluated in this article, or claim that may be made by its manufacturer, is not guaranteed or endorsed by the publisher.
