# Supplementary figures and images for: The pharmacokinetics and pharmacodynamics of esomeprazole in sheep after intravenous dosing
Source: Front Vet Sci. 2023 May 5;10:1172023. doi: 10.3389/fvets.2023.1172023 (PMC10196163; doi:10.3389/fvets.2023.1172023)

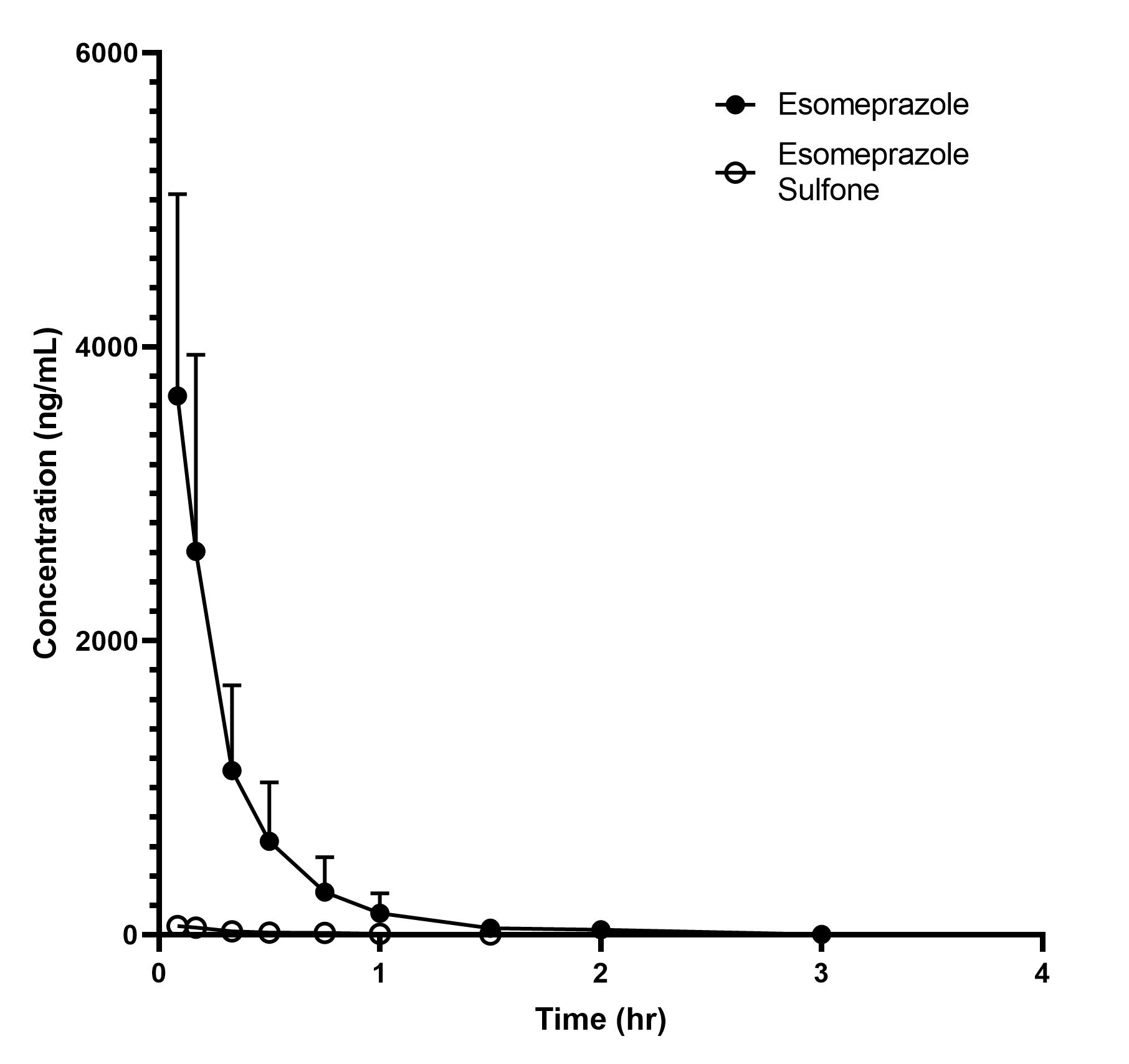

Supplement: Supplementary file 1 [file Image_1.JPEG]
